# Exploring Temporal and Spatial Characteristics of Serious Adverse Event Reports Following COVID-19 Bivalent Vaccines

**DOI:** 10.21203/rs.3.rs-6096098/v1

**Published:** 2025-03-03

**Authors:** Yiming Li, Wei Tao, Yifang Dang, Yong Chen, Cui Tao

**Keywords:** COVID-19, Vaccine, COVID-19 Vaccine, COVID-19 Bivalent Vaccine, Vaccine Adverse Event Reporting System, Adverse event following immunization

## Abstract

**Background:**

To mitigate the spread of variants such as Omicron in COVID-19 pandemic, the development and utilization of COVID-19 bivalent vaccines have become essential. However, an expected subset of individuals may experience serious adverse events (AE) after receiving the COVID-19 bivalent vaccine.

**Methods:**

In this research, we conducted an in-depth analysis of data obtained from the Centers for Disease Control and Prevention (CDC) and the Vaccine Adverse Event Reporting System (VAERS) to evaluate the safety of COVID-19 bivalent vaccines administered between 9/15/2022 and 9/1/2023. The Standard Federal Regions were used for region partitions. To broaden our understanding of post-vaccination AE, we performed temporal analysis to investigate the trends of Top 10 reported AE in all serious adverse event reports. We also examined the similarity of AE across diverse regions within the United States.

**Results:**

Our findings indicated that a relatively stably decreasing trend was observed over time, with four peaks in December 2022, February 2023, Mar 2023 and April 2023. In terms of spatial analysis, the middle and northern regions exhibited higher rates of reported AEs associated with COVID-19 bivalent vaccine. An obvious similar pattern of AE is observed across regions (III, IV, V, VI, VII).

**Conclusion:**

Overall, our research underscores the ongoing need for vigilant post-licensure vaccine monitoring, emphasizing the continuous surveillance and analysis essential for upholding the safety and effectiveness of COVID-19 bivalent vaccines.

## Plain Language Summary

Understanding Adverse Events from the Serious Cases Following COVID-19 Bivalent Vaccinations

## Introduction

The COVID-19 pandemic, caused by the novel coronavirus SARS-CoV-2, emerged as one of the most significant global health crises in recent history. As of November 14, 2023, the reported numbers from the World Health Organization (WHO) showed that there have been more than 771 million cases and nearly 7 million deaths globally and more than 103 million cases and over 1 million deaths in the United States due to COVID-19[1]. With its rapid and extensive spread, the pandemic left a profound negative impact on societies, economies, and healthcare systems worldwide[2]. Beyond the immediate threat to human lives, COVID-19 posed multifaceted challenges[3]. Not only can COVID-19 cause physical symptoms such as cough, fever, loss of taste and smell, and death, but it also exposes people to psychological conditions like sleep difficulties, anxiety, depression, and suffering from prolonged sickness effects [4], [5], [6], [7], [8], [9].

In response to this unprecedented crisis, the development and deployment of effective vaccines emerged as a pivotal strategy to control the spread of the virus and mitigate its side-effects [10], [11], [12], [13], [14]. While the existing COVID-19 vaccines have played a crucial role in curbing the pandemic, considerable adverse events (AEs) within its first two doses and ongoing mutation of the virus has raised concerns about the long-term effectiveness of COVID-19 vaccines [15]. To address this challenge, the development of a COVID-19 bivalent vaccine has gained attention as a potential solution [16], [17], [18]. A Bivalent vaccine, designed to target multiple variants or strains of the virus simultaneously, holds promise in bolstering immunity and increasing protection against evolving threats[19]. The bivalent vaccines were able to provide additional protection than the monovalent vaccines[20]. Several COVID-19 bivalent vaccines were developed and widely distributed, offering hope in the battle against the pandemic. These vaccines have proven to be an essential tool in reducing the transmission of the virus and preventing severe illness and death[21]. Both Pfizer and Moderna have bivalent vaccines targeting the original COVID-19 virus and the Omicron BA.4 and BA.5 strains were developed and authorized to different age groups [22], [23].

Despite the underlying effectiveness of COVID-19 bivalent vaccines, the incurred AEs were also gaining the authorities’ attention [24], [25]. Relevant data sources contribute to the reports of AEs within COVID-19 bivalent vaccine. For example, the Vaccine Adverse Event Reporting System (VAERS) is a national surveillance program in the United States that collects and analyzes information about AEs or side effects that occur after vaccination [12], [16]. It provides a mechanism for healthcare professionals, vaccine manufacturers, and the public to report and monitor adverse events following immunization. Hause et al. presents early safety findings for bivalent COVID-19 booster vaccination in individuals aged ≥ 12 years by using VAERS reports during August 31–October 23, 2022 [26]. The AEs reported are similar to those previously described for monovalent vaccine booster doses [26]. The findings suggest that AEs following bivalent booster doses are less common and less serious than the health impacts associated with COVID-19 illness, supporting the safety of bivalent booster vaccination [26]. Jacob et al. conducted a study examining hematologic adverse events associated with the new formulations of the Pfizer-BioNTech and Moderna bivalent COVID-19 Booster vaccines approved on August 31, 2022 [27]. Through VAERS, 55 reports of hematologic events were identified, with 3 potential cases of immune thrombocytopenia (ITP) and 1 case of vaccine-induced immune thrombotic thrombocytopenia (VITT) [27]. While the overall rate of adverse hematologic events was low (1.05 per 1,000,000 doses), the study emphasizes the importance of continued safety monitoring, especially with the expansion of vaccine use and authorization of new formulations [27]. Moro et al. carried out a study to assess the safety of bivalent mRNA COVID-19 vaccination in pregnant individuals by analyzing VAERS reports [28]. The study found that the safety profile of bivalent mRNA COVID-19 vaccination in pregnant persons was comparable to that observed for monovalent mRNA COVID-19 booster vaccination (3rd and 4th dose) in pregnant individuals based on VAERS reports [28]. AEs in pregnant persons and infants were generally consistent with known pregnancy-related complications and were comparable between bivalent and monovalent mRNA COVID-19 vaccination [28]. Additionally, the Centers for Disease Control and Prevention (CDC) administration data offers a detailed record of the number of vaccines administered within specific time frames and geographical regions, providing a nuanced understanding of the vaccine rollout [29]. The integration of CDC administration data and VAERS data allows us to align the occurrence of adverse events with the precise timing and geographic locations of vaccine administrations.

However, very few studies have paid attention to the temporal and spatial characteristics of the AE reports following COVID-19 bivalent vaccines. The temporal analysis of adverse events AEs following COVID-19 bivalent vaccination holds significant importance in several dimensions. Firstly, by scrutinizing the temporal patterns of AEs over the study period, we gain the ability to identify potential trends, peaks, or fluctuations in AE reporting. This temporal granularity is essential for detecting emerging safety concerns promptly, facilitating timely interventions, and ensuring swift responses to any unexpected patterns that may arise. Moreover, the temporal analysis allows us to correlate specific time periods with the occurrence of AEs, enabling a nuanced understanding of the factors influencing reporting dynamics. For instance, it helps discern whether certain AEs are more prevalent during particular seasons or coincide with specific vaccination campaigns, providing valuable insights for refining vaccination strategies and optimizing public health efforts. Additionally, the majority of AEs are preventable [30]. Understanding the temporal evolution of AEs contributes to the ongoing assessment of vaccine safety profiles, aiding regulatory agencies, healthcare providers, and policymakers in making informed decisions. On the other hand, the spatial examination enables us to identify potential clusters or disparities in AE reporting, shedding light on regional differences in vaccine safety profiles, which can have implications for healthcare resource allocation and targeted surveillance efforts. Overall, the combined temporal and spatial analyses not only serve as crucial components for ensuring the timeliness of interventions and enhancing our comprehension of the dynamic nature of AE reporting but also support more effective public health decision-making by providing comprehensive insights into the temporal and geographical dimensions of vaccine safety.

Therefore, in this study, our objective is to perform a thorough examination of the temporal and spatial aspects of AEs originating from serious reports submitted to VAERS following COVID-19 bivalent vaccination. The paper is structured as follows: we commence with descriptive analyses of vaccine administration and VAERS data, exploring factors like gender, age, and manufacturer, with a specific emphasis on serious reports. Following this, we delve into our investigation of the temporal variation of AEs, providing a comprehensive analysis. By modeling weekly reported AEs relative to administration, we accurately evaluate temporal patterns, uncovering associations between time periods and AEs. In addition to the temporal analysis, we conduct a spatial analysis using the BioWordVec_PubMed_MIMICIII embedding model. This method enables us to create meaningful vectors capturing the subtleties of symptom compositions, allowing us to scrutinize the serious reporting rates of adverse events in various regions across the United States. Overall, our approach integrates advanced embeddings, semantic similarity, and temporal modeling, offering comprehensive insights into AE serious reporting following COVID-19 bivalent vaccinations.

## Method

### Data source

We gathered VAERS records related to adverse events post-COVID-19 bivalent vaccination between 9/15/2022 and 9/1/2023. These records can be accessed through the VAERS website at https://vaers.hhs.gov/data/datasets.html. They are distributed across three CSV files, namely VAERSDATA.CSV, VAERSVAX.CSV, and VAERSSYMPTOMS.CSV, each classified by year. The VAERSDATA.CSV file includes demographic data, date of vaccination and AEs, descriptions of symptoms, history of allergies, and serious outcomes. Information about the vaccine’s type and manufacturer for every adverse event is detailed in the VAERSVAX.CSV. The VAERSSYMPTOMS.CSV delineates symptoms for each adverse event, corresponding to the Preferred Term (PT) from the MedDRA terminology. All three files are linked using the ‘VAERS_ID’ as the primary key.

Moreover, we gathered data on COVID-19 bivalent vaccine administration from the CDC’s COVID Data Tracker for the same time frame. This tracker, overseen by the CDC, offers current insights into COVID-19 bivalent vaccine distribution throughout the U.S [29]. It details the quantity of vaccine doses both distributed and given, and offers specifics by state, demographic category, and type of vaccine [29].

### Descriptive analysis

We organized the VAERS entries based on gender, age, and vaccine manufacturers as follows. Gender was designated as male, female, or unspecified. We segmented the reports into six age brackets using CDC-advised age demarcations (5, 12, 18, 65), with “unspecified” being used for entries with missing age data. The vaccine manufacturers included the predominant COVID-19 bivalent vaccine brands available, namely Pfizer/BioNTech, and Moderna. We excluded records (N = 13) that combined Pfizer and Moderna vaccines, as it was indeterminable which vaccine might have caused the AEs based on the provided details.

A report was deemed serious if it indicated any of the subsequent outcomes: fatality; events deemed life-threatening when they occurred; admission to a hospital or extension of a current hospital stay; enduring or substantial disability/impairment; a birth defect or congenital anomaly; or an event of medical significance based on professional medical evaluation [31]. We labeled such instances as “serious reports” if the associated fields showed any conditions like “DIED”, “L_THREAT” (indicating life-threatening events), “ER_VISIT” (denoting an emergency room visit), “X_STAY” (indicative of a hospital stay or its extension), or “DISABLE” (reflecting chronic or major disability/impairment). To delve deeper into the serious adverse reactions (SAEs) linked with the COVID-19 bivalent vaccines, we further scrutinized the serious reports, placing emphasis on the distribution of cases by gender, age bracket, and vaccine manufacturers.

### Temporal analysis

In our research, we carried out an in-depth temporal analysis to study the weekly serious reporting rate of AEs (Eq. (1)) following COVID-19 bivalent vaccination. We evaluated and ranked the top 10 AEs present in serious case reports over time.


(1) $$\text{Reporting}\;\text{rate}\;\text{of}\;\text{AEs}=\frac{\text{number}\;\text{of}\;\text{reported}\;\text{AEs}}{\text{number}\;\text{of}\;\text{vaccinations}\;\text{administered}}$$

### Spatial analysis

We carried out a geographical evaluation of the case and serious case reporting rates across each U.S. state. For consistent regional categorization of states, we referred to the Standard Federal Regions outlined in the Circular A-105 issued by the Office of Management and Budget in April 1974 (see Supplementary Table S1) [32].

Moreover, we conducted a statistical examination of symptom distribution across each region, creating vectors that consider both the AEs and their occurrence rates. To gauge the resemblance of symptom patterns across regions, we leveraged the BioWordVec_PubMed_MIMICIII embedding model. This model, rooted in the word2vec algorithm, is tailored to enhance biomedical word representations. It benefits from subword details and integrates the extensive PubMed and MIMIC-III datasets to produce embeddings that grasp the intricate interpretations of biomedical terms [33], [34].

## Results

**Figure 1:**
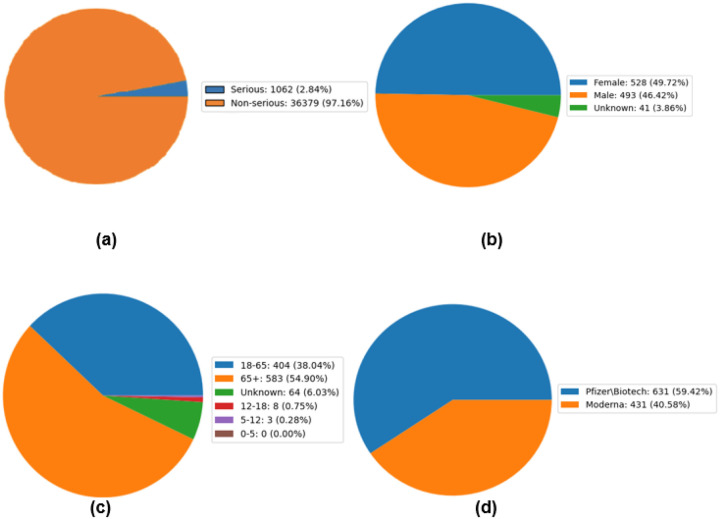
Descriptive results of 2022–2023 VAERS COVID-19 bivalent data. (a) the proportion of serious reports of the total COVID-19 bivalent vaccination reports. (b) the gender distribution of these serious reports. (c) the age distribution of these serious reports. (d) the manufacturer distribution of these serious reports.

Figure 1 provides a comprehensive breakdown of the SAE reports analyzed in this study. Out of the 37,441 reports examined, 1,062 were categorized as SAEs. Among these, 528 were filed by female patients, and 493 by male patients. The largest proportion of serious VAERS reports came from the senior age group (65+), making up 54.90% of the total reports, followed by individuals aged 18–65, who submitted 404 serious AE reports. In terms of vaccine manufacturers, Pfizer had the highest number of serious VAERS reports, totaling 631 cases, while Moderna had 431 cases.

Among the SAE reports, a total of 8,723 non-unique AEs and 1,907 unique AEs were identified. The most commonly reported AEs included death (N = (250)), dyspnoea (N = 115), and COVID-19 (N = 107). The most frequent SOCs reported were Investigations (N = 2,827), General disorders and administration site conditions (N = 1,093), Nervous system disorders (N = 934),Respiratory, thoracic and mediastinal disorders (N = 622), and Surgical and medical procedures (N = 507).

### Temporal Analysis

**Figure 2:**
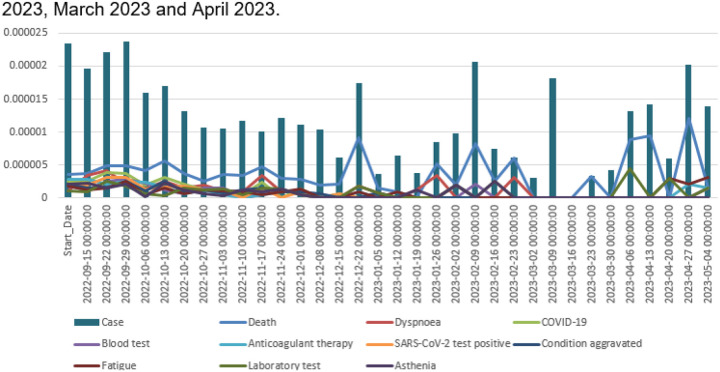
Serious Reporting Rate and Serious Case Top 10 AEs Reporting Rate Over Time

In Fig. 2, we present the trend in the proportion of reporting rates and the top 10 reported AEs associated with serious reports in VAERS. Our analysis reveals that the top 10 reported AEs include death, dyspnea, COVID-19, blood test, anticoagulant therapy, SARS-CoV-2 test positive, condition aggravated, laboratory test, and asthenia. Generally, we observe a relatively stable decreasing trend, particularly prior to December 2022. Notably, four local peaks are evident in December 2022, February 2023, March 2023 and April 2023.

### Spatial Analysis

**Figure 3:**
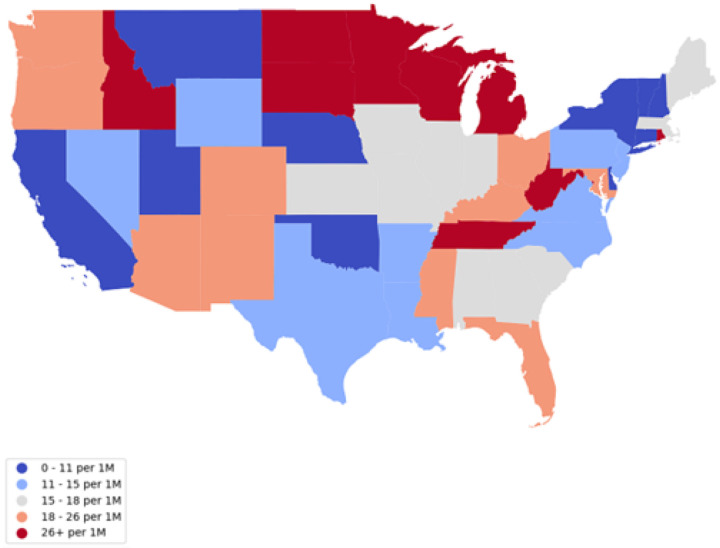
VAERS serious reporting rate by State

In Fig. 3, we observe the VAERS serious reporting rate. Remarkably, South Dakota (SD), West Virginia (WV), Idaho (ID), Tennessee (TN), Rhode Island (RI), Wisconsin (WI), Michigan (MI), and North Dakota (ND) emerge as the states with serious reporting rates exceeding 30μ. Among them, South Dakota (SD) takes the lead with the highest reporting rate, reaching a substantial 83.9μ.

**Figure 4:**
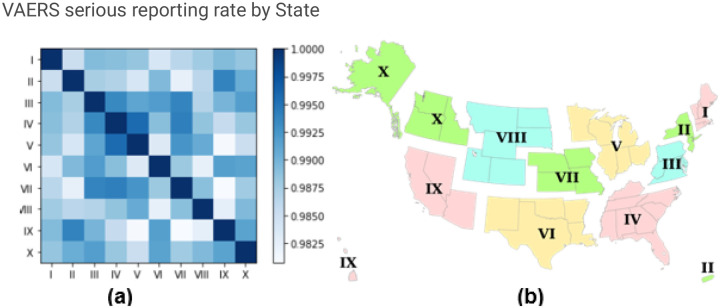
(a) The heatmap of the similarity of AEs for serious reports between regions (b) Standard Federal Regions [32]

Figure 4 presents a heatmap illustrating the similarity of AEs in serious reports among different regions within the United States. The analysis unveils that the similarity between all regions surpasses 0.98, denoting a remarkable similarity in reported AEs. Specifically, Region IV and Region V stand out with the highest level of similarity, marked by an exceptional value of 0.99561727, indicating a substantial correlation in the AEs reported between these regions. On a broader scale, as seen in the darker boxes within Fig. 4, it appears that Region III, IV, V, VI and VII cluster together, exhibiting higher similarity within this group.

## Discussion

Most AEs are preventable [30]. Our research has yielded compelling insights that reveal the temporal and spatial characteristics of the serious AE cases following COVID-19 bivalent vaccines and emphasize the clinical relevance. We notably identified peaks in serious adverse event reports in December 2022, February 2023, March 2023, and April 2023. The observed peak reflects a complex interplay of many factors, such as public attitudes, evolving variants, etc. during that specific period. The CDC’s recommendation for an updated bivalent booster vaccine in September 2022 aimed to enhance protection against emerging Omicron subvariants [35], [36]. However, by the end of 2022, it is published that only 27.1% of vaccinated adults had received the booster, as revealed by interviews and surveys [37], [38]. Negative results about COVID-19 vaccines were one of the common reasons for peaks in serious adverse event reporting. Second, rampancy of SARS-CoV-2 omicron XBB variant (eg, XBB.1.5 and XBB.1.9) starting Feb 2023, is another contributor to the increase in the serious adverse event reporting in the Spring 2023 [39]. Moreover, the gradual adoption of the COVID-19 bivalent vaccine, leading to its full utilization by May 2023, can also account for the peak in adverse events during these months [40].

Our spatial analysis reveals obvious patterns in serious case reporting rates across the United States. Specifically, the north and middle regions exhibit notably higher reporting rates compared to the southeast and southwest regions, which shows the similar characteristics of distribution as the COVID-19 vaccine [16]. This phenomenon may stem from multiple factors, including variations in population density, vaccination coverage, and differences in healthcare access and reporting systems among regions [16]. In the context of COVID-19 bivalent vaccine AEs from serious reports, Regions III to VII exhibit a much higher degree of similarity.

It is important to note that we identified 26 cases of Guillain-Barre syndrome and 4 cases of autoimmune hepatitis in this study. The association between vaccinations and autoimmune diseases is an area of active research and debate. One of the key hypotheses proposed to explain this immunological association is epitope mimicry, akin to mechanisms seen in infections [41]. This theory suggests that an antigen introduced by the vaccine might bear structural similarities to self-antigens [41]. Consequently, the immune response triggered by the vaccine antigen could inadvertently target other host cells expressing the structurally similar self-antigen [41]. Another potential mechanism is bystander activation, which is an antigen non-specific process that activates autoreactive T cells [41], [42]. However, it’s important to note that these hypotheses are based on theoretical considerations, and more research is needed to establish a definitive link between COVID-19 booster vaccinations and autoimmune diseases.

Our examination of AEs after COVID-19 bivalent vaccination excels existing research in several ways. To begin, unlike prior studies, we made use of both vaccine administration data and VAERS data. This method enabled us to assess the frequency of AE reporting rather than relying solely on absolute values. The use of both datasets offers the advantage of presenting a more precise depiction of AEs associated with COVID-19 bivalent vaccines. Additionally, we conducted an analysis spanning almost three years to establish a more comprehensive and persuasive conclusion. Most studies have been conducted over shorter periods, limiting their scope and reliability. In contrast, our study investigated AEs following COVID-19 bivalent vaccination between 2022 and 2023, enabling us to evaluate the long-term safety profile of these vaccines. This approach also provides a more dynamic and unbiased understanding of the risks linked to COVID-19 bivalent vaccines. Our method allowed us to identify the most prevalent AEs associated with COVID-19 bivalent vaccines and their underlying mechanisms. This information can assist healthcare providers in improving the management and treatment of AEs associated with COVID-19 bivalent vaccines. Moreover, we harnessed the capabilities of an embedding model to examine symptom similarities across diverse regions. This model can handle complex word structures and enhance the representation of rare or previously unseen terms by considering subword units. This approach enables us to extract more precise and meaningful insights from biomedical texts, facilitating various applications such as biomedical information retrieval, named entity recognition, and text classification. Overall, this embedding model serves as a valuable resource for advancing biomedical text analysis and expediting biomedical research. Last but not least, we conducted not only a horizontal comparison in terms of temporal and spatial analysis for AEs in COVID-19 bivalent vaccine, but also a longitudinal comparison with the AE analysis COVID-19 vaccines performed by Li et al., allowing us to perform a more comprehensive analysis of AEs associated with the COVID-19 bivalent vaccine [16].

Nevertheless, it is essential to acknowledge the limitations of our study. Firstly, the data’s quality is insufficient because we could not access certain administration data due to its unavailability in the CDC database, which could have enriched our analysis. Secondly, we omitted cases in which patients received a combination of Pfizer and Moderna vaccines, resulting in selection bias. Unfortunately, this also meant that we could not filter out such cases in the administration data. However, it’s important to note that the number of individuals receiving mixed doses per week was minimal compared to the overall number of vaccinations, which mitigated the potential impact of this bias. VAERS is a valuable tool for monitoring the safety of vaccines, but it also has several limitations. One major issue is underreporting, where not all AEs are reported to VAERS. This can occur due to a variety of reasons, including lack of awareness among healthcare providers and the public, uncertainty about whether an AE is vaccine-related, and the perception that reporting is time-consuming or unnecessary. As a result, VAERS data may not capture the full extent of vaccine-related AEs, leading to potential gaps in surveillance and analysis. Furthermore, reports sent to VAERS may contain incomplete information, such as missing details about the individual’s medical history or the timeline of events surrounding the AE. This can make it challenging to fully understand the context of the reported event and its potential relationship to the vaccine. Inaccuracy is another issue, as reports may include errors or inaccuracies in the description of the AE, the vaccine administered, or the individual’s demographic information. These inaccuracies can lead to difficulties in accurately assessing the true nature and scope of vaccine-related AEs. Coincidental events, which occur by chance and are not caused by the vaccine, can also be reported to VAERS. Distinguishing between true vaccine-related AEs and coincidental events can be challenging, especially without comprehensive medical evaluation and follow-up. Additionally, reports sent to VAERS may include unverified information, such as second-hand or anecdotal reports. Without proper verification and validation, this information may not be reliable for making informed decisions about vaccine safety. Despite these limitations, we believe that our study offers a comprehensive analysis of adverse events following COVID-19 bivalent vaccination.

## Conclusion

Post-licensure vaccine surveillance plays a pivotal role in biomedical research, safeguarding the safety and effectiveness of vaccines. The VAERS serves as an essential initial step in this process by providing insights into adverse events following immunization. In our study, we applied VAERS data in conjunction with CDC administration data to conduct a comprehensive secondary analysis of AEs linked to COVID-19 bivalent vaccines. Our analysis covered both the temporal and spatial dimensions, offering us a holistic understanding of the vaccines’ safety profile.

To further advance our research, we intend to apply advanced statistical methodologies to uncover potential correlations between AEs and COVID-19 bivalent vaccinations. This approach aims to provide deeper insights into the factors contributing to AEs and facilitate more targeted interventions to improve vaccine safety.

In summary, our study underscores the critical role of ongoing post-licensure vaccine monitoring and emphasizes the necessity for continuous surveillance and analysis to uphold the safety and effectiveness of COVID-19 bivalent vaccines.

## Data Availability

The datasets are available at https://vaers.hhs.gov/data/datasets.html.

## What was the study about?

Serious adverse event reports involve life-threatening incidents or substantial disabilities and consequences. This study explored serious adverse event reports after people received COVID-19 bivalent vaccines. We used such reports from the Vaccine Adverse Event Reporting System (VAERS) to examine the temporal trends and spatial distribution of adverse event reporting.

## Why did we do this study?

While bivalent vaccines are crucial in controlling the pandemic, it’s important to monitor their safety. Understanding the temporal and spatial patterns of reported adverse events (AEs) can promote health officials to respond more efficiently and develop vaccines even safer.

## How did we do this study?

We collected data from VAERS, focusing on reports between September 2022 and September 2023. We analyzed these reports based on age, gender, and vaccine manufacturer. We looked for trends over time and differences across various regions in the United States.

## What did we find?

Serious cases were more reported by older adults (65+), with a similar number of reports from men and women. The most common AEs in the serious adverse event report included death, dyspnea, COVID-19. Our findings showed a consistently declining trend over the observed period. The middle and northern regions had higher serious report rates of than others. The reported AEs were very similar across central and eastern regions.

## What do these results mean?

The study helps us understand how AEs from serious adverse event reports following COVID-19 bivalent vaccines are distributed across time and different regions. This information is crucial for ongoing vaccine safety monitoring. Knowing where and when AEs are reported can help health officials take timely action and ensure vaccines are as safe as possible.

## What’s next?

Future research should continue to explore the reasons behind these trends and work on improving the methods for analyzing vaccine safety.
